# The knowledge, attitude and practice of community people on dengue fever in Central Nepal: a cross-sectional study

**DOI:** 10.1186/s12879-022-07404-4

**Published:** 2022-05-12

**Authors:** Parbati Phuyal, Isabelle Marie Kramer, Ulrich Kuch, Axel Magdeburg, David A Groneberg, Mandira Lamichhane Dhimal, Doreen Montag, Harapan Harapan, Edwin Wouters, Anjani Kumar Jha, Meghnath Dhimal, Ruth Müller

**Affiliations:** 1 Institute of Occupational Medicine, Social Medicine and Environmental Medicine, Goethe University, Frankfurt am Main, Germany; 2 Institute of Environment and Sustainable Development, University of Antwerp, Antwerp, Belgium; 3 Policy Research Institute (PRI), Kathmandu, Nepal; 4 Global Institute for Interdisciplinary Studies (GIIS), Kathmandu, Nepal; 5 Wolfson Institute of Population Health, Queen Mary University of London, London, UK; 6 Medical Research Unit, School of Medicine, Universitas Syiah Kuala, Banda Aceh, Indonesia; 7 Tropical Disease Centre, School of Medicine, Universitas Syiah Kuala, Banda Aceh, Indonesia; 8 Department of Microbiology, School of Medicine, Universitas Syiah Kuala, Banda Aceh, Indonesia; 9 Department of Sociology, University of Antwerp, Antwerp, Belgium; 10 Nepal Health Research Council, Ramshah Path, Kathmandu, Nepal; 11 Unit Entomology, Institute of Tropical Medicine, Antwerp, Belgium

**Keywords:** *Aedes*, Awareness, Epidemics, Public health

## Abstract

**Background:**

Since 2006, Nepal has experienced frequent Dengue fever (DF) outbreaks. Up to now, there have been no knowledge, attitude and practice (KAP) studies carried out on DF in Nepal that have included qualitative in-depth and quantitative data. Thus, we aimed to explore and compare the KAP of people residing in the lowland (< 1500 m) and highland (> 1500 m) areas of Nepal.

**Methods:**

A cross-sectional mixed-method study was conducted in six districts of central Nepal in September–October 2018 including both quantitative (660 household surveys) and qualitative data (12 focus group discussions and 27 in-depth interviews). The KAP assessment was executed using a scoring system and defined as high or low based on 80% cut-off point. Logistic regression was used to investigate the associated factors, in quantitative analysis. The deductive followed by inductive approach was adopted to identify the themes in the qualitative data.

**Results:**

The study revealed that both the awareness about DF and prevention measures were low. Among the surveyed participants, 40.6% had previously heard about DF with a significantly higher number in the lowland areas. Similarly, IDI and FGD participants from the lowland areas were aware about DF, and it’s associated symptoms, hence they were adopting better preventive practices against DF. The findings of both the qualitative and quantitative data indicate that people residing in the lowland areas had better knowledge on DF compared to people in highland areas. All IDI participants perceived a higher chance of increasing future dengue outbreaks due to increasing temperature and the mobility of infected people from endemic to non-endemic areas. The most quoted sources of information were the television (71.8%) and radio (51.5%). Overall, only 2.3% of the HHS participants obtained high knowledge scores, 74.1% obtained high attitude scores and 21.2% obtained high preventive practice scores on DF. Among the socio-demographic variables, the area of residence, educational level, age, monthly income, SES and occupation were independent predictors of knowledge level, while the education level of the participants was an independent predictor of the attitude level.

**Conclusions:**

Our study found a very low level of knowledge and insufficient preventive practices. This highlights an urgent need for extensive dengue prevention programs in both highland and lowland communities of Nepal.

**Supplementary Information:**

The online version contains supplementary material available at 10.1186/s12879-022-07404-4.

## Background

Dengue fever (DF) is a mosquito-borne viral disease caused by four serotypes of the dengue virus (DENV-1, DENV-2, DENV-3, and DENV-4) [1]. DF has become a major international public health concern with an estimated 10,000 deaths and 100 million symptomatic infections per year in over 128 countries, predominantly in Asia, followed by Latin America and Africa [2–5]. It is mainly transmitted to humans by the mosquito vectors *Aedes aegypti* and *Aedes albopictus*, which have spread to tropical and sub-tropical regions around the globe, predominantly in urban and semi-urban areas [6]. The distribution of such vector-borne diseases is determined by a complex set of environmental and social-demographic factors [7, 8]. Climate change along with rapid landscape and demographic changes is already changing the environment of Himalayan countries, such as Nepal, causing the shifting of disease vectors and disease transmission from tropical to temperate and highland areas [9, 10]. Furthermore, warming in the Himalayas is reported to be greater than the global average temperature rise (0.06 °C/year) indicating that the Himalayas are more sensitive and vulnerable to climate change [11]. Accordingly, the expansion of dengue and chikungunya cases and their vectors in the countries of the Hindu Kush Himalayan region, including Nepal, has been documented [12].

The first DF case in Nepal was reported in 2004 [13], while the first dengue outbreak was reported from the lowland areas in 2006 [14] with circulation of all four dengue serotypes [15]. Since 2006, Nepal has continued to experience DF outbreaks with increasing cases from the lower altitudes up to the hilly regions, with a significant impact on public health [12, 15–24]. In 2019, Nepal experienced a large dengue outbreak with more than 17,000 reported cases from the lowland areas [< 1500 m above mean sea level (amsl)] to the highland areas (> 1500 m amsl) including some areas which were not previously reported as being dengue-endemic [16, 25]. However, the majority of cases until 2018 have been reported from the lowland areas (< 1500 m) which are densely populated [12, 16, 20] with known distribution of *Aedes* vectors, i.e., *Aedes albopictus* and *Aedes aegypti* [20, 26]. The frequent outbreaks of DF and the rising number of dengue cases in Nepal suggest that the vector control efforts are probably ineffective or insufficient and are conducted exclusively only as part of an emergency response to outbreaks [27]. In the meantime, the vaccine development against DF has made remarkable progress in recent years, however, the vaccines are unavailable in Nepal and also do not protect against all serotypes of DF [5, 28, 29]. Furthermore, an individual can be infected with dengue several times, which eventually increases the risk of severe dengue infection [30]. In the absence of an efficacious vaccine and specific antiviral treatment, vector prevention and control strategies have helped to minimize the increase in dengue frequency and the severity of dengue epidemics [31]. Community participation may offer a more cost-effective approach and, therefore, provide more sustainable dengue reduction interventions [32]. Meanwhile, in general human behavior change communication (BCC) is one of the strategies currently adopted for reducing the vector population and dengue virus transmission [33, 34]. However, it is important to consider that the diverse ethnic groups from distinct socio-economic and cultural backgrounds reside in the different altitudinal gradients of Nepal [35]. Prior to 2019, several sporadic outbreaks of DF were only reported from the lowlands, while the highland regions were considered as non-dengue endemic areas [16, 21, 36–38]. Thus, in order to improve and design sustainable public health interventions for dengue throughout the different altitudinal regions of Nepal, with people having different socio-economic and cultural backgrounds, it is essential to recognize and understand the people’s knowledge, attitude and practices (KAP) on dengue virus and its vectors [39, 40]. While several KAP studies have been conducted in Nepal, these were limited to specific dengue-endemic areas [41, 42] or only focused on dengue-infected people [43]. Furthermore, none of the previous studies were conducted in both the highland and lowland areas except for one study by Dhimal et al. [40] which was focused on quantitative data only. Thus, this present mixed-method study aimed to assess and compare the KAP among community groups residing in lowland and highland areas, applying both quantitative and qualitative methods. The purpose of using both methods was to triangulate the study outcomes, gaining an in-depth understanding of the general community and the public health professionals who were presumed to be in better receipt of health information.

## Methodology

### Study design and site description

**Fig. 1:**
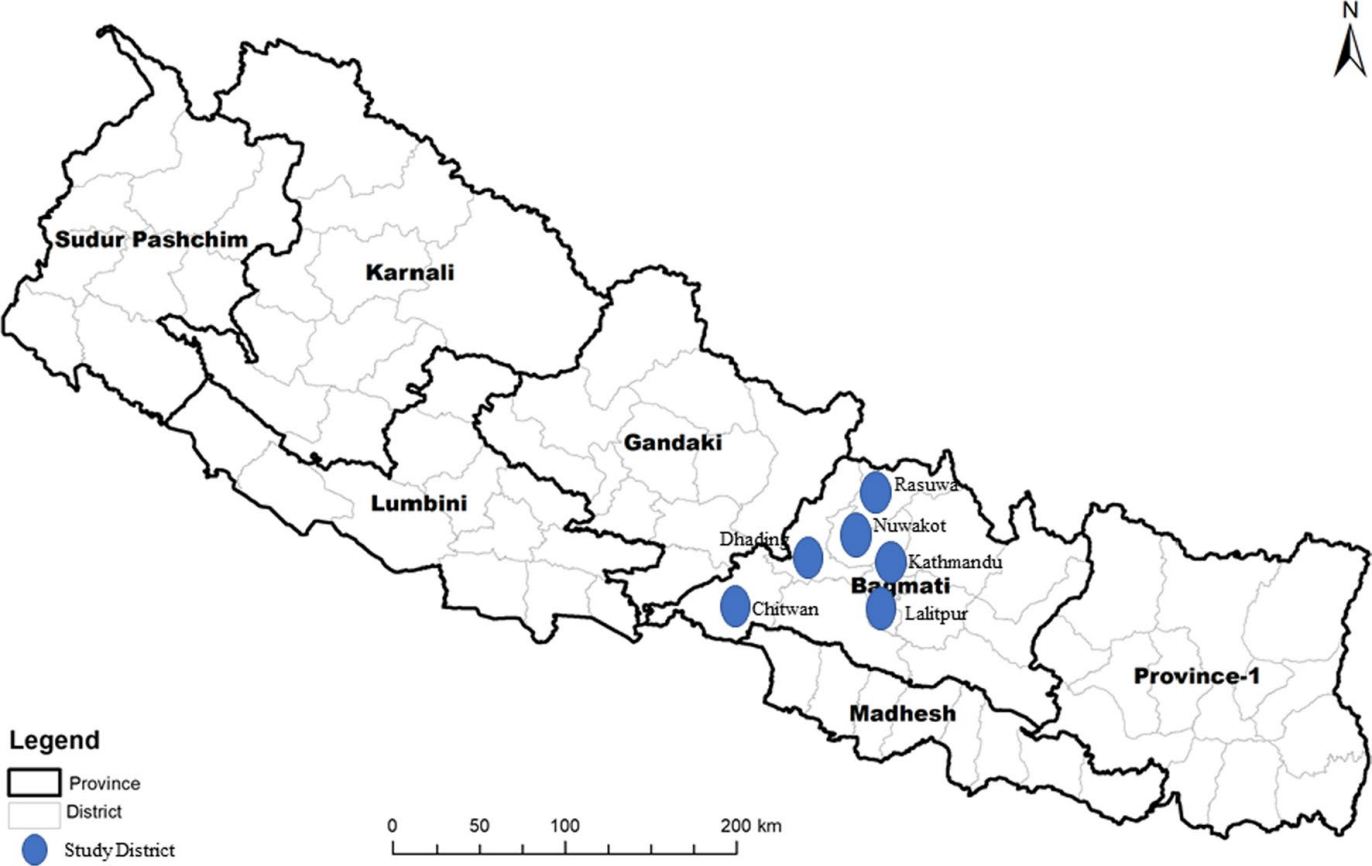
Study areas (blue) in Bagmati province (province-2), Nepal

In September and October 2018, a cross-sectional mixed-method (quantitative and qualitative) study was conducted in the lowland and highland communities of Central Nepal using household surveys (HHS), focus group discussions (FGDs) and in-depth interviews (IDIs). Central Nepal covers all types of physiographical regions from the lowlands of Terai and Siwalik, to middle mountains and high mountain regions of up to 7276 m amsl [44]. Initially, six administrative districts (Chitwan, Dhading, Kathmandu, Lalitpur, Nuwakot and Rasuwa) of Central Nepal (Bagmati Province), extending along an altitudinal range from 100 m to 2100 m amsl were selected (Fig. 1). Subsequently, the parts of the study area below 1500 m amsl (Chitwan, Dhading, Lalitpur and Kathmandu) were categorized as lowland and those above 1500 m amsl (Nuwakot and Rasuwa) as highland areas based on a previous study [40]. The lowland areas are predominantly urban areas compared to rural highland areas with tropical to subtropical climates, while the highland areas experience a temperate to alpine climate [40, 45–47]. Whilst several sporadic DF outbreaks have been reported from the lowland areas since 2006, no cases of DF were reported from the highland areas prior to 2019 [12, 16, 20, 21, 36–38]. The distribution of the *Aedes* vector is more common in lowland areas (< 1500 m amsl), while it is rarely observed or is less common in the highland areas (> 1500 m amsl) [20, 26, 48]. This research uses a concurrent mixed-methods design. Qualitative and quantitative data collection were carried out in parallel.

### Study variables

We collected data on (a) demographic information (the age, education, occupation, marital status, income, ethnicity and type of residence of the participants); (b) whether or not they, their family members or their neighbors had already suffered from DF; (c) knowledge about DF (symptoms, vectors, management and prevention); (d) attitude towards DF; and (e) preventive and control practices against DF, i.e., methods used to reduce breeding sites and to reduce human mosquito contact (bed nets, repellents, and window screens). Participants were also asked about their sources of information on DF. The asset index from Filmer and Lritchett [49] was adapted to measure and categorize the socio-economic status (SES) of the participants, wherein the 1st quartile was assigned as the poorest and the 5th quartile as the least poor. Similarly, the contents discussed in the FGDs and IDIs included knowledge and awareness on dengue, its vector and transmission, the perceived risk towards DF and the prevention and control practices undertaken against DF.

### Household questionnaire survey

The unit of sampling used for this study was the household; this was defined as all those eating from the same cooking pot or using the same cooking hearth [50, 51]. Sample size was calculated assuming 50% population having knowledge with 95% confidence level and 5% allowable error. After adding 10% for non-responses, our sample in each district became 106 and the total sample size (6 districts of Central Nepal) became 636. This number was rounded up to 660 for convenience. Therefore, we targeted 660 households (110 from each district) for the study employing a systematic random sampling method. For this, five vector collection sites [52, 53] were randomly selected from each district for the KAP survey. Thereby, all households located within a 50 m radius of each site were listed by performing a social mapping exercise and, subsequently, 22 households were selected from each site by following the systematic random sampling method [54]. For this sampling method, we first calculated the sampling interval by dividing the total number of households in each site (by social mapping) by the number of households, we targeted to sample, i.e., n = 22. Thus, 22 households were selected from each vector collection site according to the sample interval. Consequently, all eligible individuals (aged 18 or above and who had not moved away or died) were listed from each selected household and one participant from that list was selected randomly to take part in the survey using the WHO-Kish method [55]. A set of validated and previously used KAP questionnaires [40] were adapted for our study. Cronbach’s alpha was used to assess the reliability coefficient [56], where the questionnaire was tested for internal consistency among 100 participants of the Chitwan district. This pretest data was not included in the final analysis. The obtained Cronbach’s alpha coefficients of the KAP domains were 0.8, 0.7 and 0.8, respectively. Here, a minimum value of 0.7 was considered to reflect an acceptable internal reliability [57]. University graduates were hired and trained for data collection, however, they were not informed about the study’s hypothesis or correct answers in order to avoid interviewer bias during data collection. Questions related to KAP were asked one-by-one, sequentially, to avoid bias.

### In-depth interviews and focus group discussions

Twelve FGDs were conducted among 96 community people of the highland and lowland areas. The FGDs consisted of 6–12 individuals per group with similar socio-economic backgrounds [58]. The FGD helps to validate the perceptions, as the group becomes a tool for reconstructing individuals’ opinions more appropriately [59]. Similarly, the IDIs were carried out using purposive sampling [60]. IDIs (n = 27) were conducted with local political leaders, community leaders, female community health volunteers (FCHVs), teachers and public health professionals. The FGDs and IDIs were conducted in the Nepali language by following the semi-structured guidelines for interviewing and the FGD; these discussions and interviews were tape-recorded. However, in two cases, the interviews were not recorded due to unexpected technical problems and only notes were prepared. The optimum number of FGDs and IDIs were determined based on the theory of saturation [58]. This means that when the information obtained from the FGDs and IDIs was repeated or when no new information was generated, no further IDIs and FGDs were carried out.

### Quantitative data analysis

The data were verified and entered using the Epi Data 3.1 Software (EpiData Association, Denmark). All quantitative data analyses were performed using the Statistical Package for the Social Sciences software (IBM SPSS Statistics for Windows, Version 24). The participant’s total KAP score about DF was calculated by assigning one score for each correct answer and zero score for each wrong answer. “Do not know” (DNN) responses were also given a zero score by considering it as a wrong answer [61]. These single scores were summed up according to the number of questions in the questionnaire to obtain a possible total score of 24 for knowledge, 6 for attitude and 21 for practice. Thus, after obtaining a single summed up value of each domain separately, participant’s levels were defined. The level of knowledge was divided into three categories: “no knowledge” (those who never heard about dengue prior to the survey), “low knowledge score” and “high knowledge score”. The last two knowledge groups were dichotomized based on an 80% cut-off point, i.e., who scored < 80% of total score (score: 1–18) as low knowledge score, while who scored ≥ 80% (Score: ≥ 19) as high knowledge score [40, 62]. The level of attitude and practice were also assessed as ‘‘high score’’ or ‘‘low score’’ based on same threshold i.e., 80% cut-off point [40, 62]. For attitude domain, 80% cut-off score was five and for practice domain, 80% cut-off score was 17. Thus, the obtained total scores that were ≥ 80% were categorized as high scores, while those < 80% were categorized as low scores based on number of questions of each KAP domain, and for the attitude and practice domain all those who had never heard about dengue before were excluded. An additional file shows the thresholds for scoring the KAP domain in more detail (see Additional file 1). The Chi-square test was used to compare the socio-demographic characteristics and the KAP level between the highland and lowland areas. The Fisher’s exact test was used when more than 20% cells have expected count less than 5. Spearman’s rank correlation (rs) was used to calculate the correlation values between the KAP scores because these were not normally distributed, as revealed by a Shapiro–Wilk normality test. The Bootstrap method was used to calculate the confidence intervals (CI) for the Spearman’s rank correlation [63] and to compare the correlations of the KAP scores between the highland and lowland areas. The logistic regression analysis (univariate and multivariate) was employed for the knowledge attitude and practice domain. All socio-demographic variables were included as explanatory variables in logistic regression analysis. Due to very few “high knowledge score” events (15 in total) obtained in knowledge domain, we could not apply a model to this variable, therefore, “high knowledge score” were excluded and the levels of knowledge, “low knowledge” vs “no knowledge” were only used as the outcome variables in logistic analysis. Similarly, the levels of attitude and practice, “high score” vs. “low score”, were used as the outcome variables in the logistic regression analysis respectively. In the next step, all explanatory factors with P ≤ 0.25 from univariate analyses were entered into the multivariate analysis [40, 62, 64]. In addition, we also ran multilevel modeling to account the variations between the clusters; this revealed no significant variation in intercepts across the clusters. Confounding factors were explored by comparing the difference between the adjusted odds ratio (aOR) in the multivariate analyses and the crude odds ratio (OR) in the univariate analyses, of a particular predictor variable on the knowledge, attitude and practice domains. All “*P*-values” were two-tailed and were considered statistically significant at “*P* < 0.05”.

### Qualitative data analysis

Qualitative data analysis was performed using the MAXQDA software. First, we transcribed the FGDs and IDIs in the Nepali language and later translated them into English. Initially based on findings from the literature, themes and sub-themes were defined and then used to create a ‘code list’. Emerging themes from the transcripts were added. In order to avoid biasness in translation, the translations were double-checked by at least two study team members. An English version of each transcript was uploaded in the MAXQDA software for analysis. With the help of this code list, the data coding and recoding of the transcripts were carried out in MAXQDA software, as used in a previous study in Nepal [65]. Subsequently, a coding guideline was developed following the deductive and inductive category assignments and, thus, the English versions of the transcripts were coded with those defined categories, accordingly. The second step was to extract all coded material per category and to summarize the material per category; a summary of the main themes is shown in Table 1. Qualitative content analysis was carried out using the deductive category assignment approach, as described in Marying [66]. We used two steps for content structuring or theme analysis, i.e., deductive followed by inductive.

**Table 1:** Summary of the main themes (Qualitative data)

Theme area probed in discussion	Key themes identified
Knowledge and awareness	Knowledge about symptoms, vector, transmission and prevention: Fever and joint pain as common signs of DF, *Aedes* (white stripes on head) responsible for transmitting DF
Attitudes towards dengue Perceived risk	Perceived high risk: concerning future epidemics
Prevention and control practices	Prevention against mosquito bites Destruction of mosquito breeding sites
Sources of Information	Through mass media: Radio, television Through social relations: Neighbors

## Results

### Characteristics of the study population in Central Nepal

Twelve FGDs were conducted with 41 males and 55 females, while 27 IDIs included 16 males and 11 females. Out of the total 660 households/individuals, 651 participants were enrolled for the questionnaire survey with a response rate of 98.6%. Among the interviewed participants, 33.5% were residents of the highland areas and 66.5% were from the lowlands (Table 2). Among the socio-demographic characteristics, age (*P* < 0.05), ethnicity (*P* < 0.001), educational level (*P* < 0.001), occupation (*P* < 0.001), and monthly income (*P* < 0.001) were significantly different between lowland and highland areas (Table 2).

**Table 2:** Socio-demographic characteristics and KAP of participants (highland vs lowland (N = 651)

Socio-demographic characteristics	Highland n (%)	Lowland n (%)	Total n (%)	*P*-value
Age group (years)				
19–29	54 (24.8)	66 (15.3)	120 (18.5)	0.016
30–44	82 (37.6)	194 (45.0)	276 (42.5)	
45–60	55 (25.2)	102 (23.7)	157 (24.2)	
> 60	27 (12.4)	69 (16.0)	96 (14.8)	
Sex				
Male	81 (37.2)	152 (35.1)	233 (35.8)	0.606
Female	137 (62.8)	281 (64.9)	418 (64.2)	
Ethnicity				
Dalit	9 (4.1)	21 (4.8)	30 (4.6)	< 0.001*
Disadvantaged Janajatis	138 (63.3)	51 (11.8)	189 (29)	
Disadvantaged non Dalit Terai caste	0	7 (1.6)	7 (1.1)	
Religious minorities	0	2 (0.5)	2 (0.3)	
Relatively advantaged Janajatis	47 (21.6)	112 (25.9)	159 (24.4)	
Upper caste	24 (11)	240 (55.4)	264 (40.6)	
Educational qualification				
Illiterate	52 (23.9)	53 (12.2)	105 (16.1)	< 0.001
Literate	92 (42.2)	121 (27.9)	213 (32.7)	
Secondary	32 (14.7)	79 (18.2)	111 (17.1)	
Higher secondary	34 (15.6)	106 (24.5)	140 (21.5)	
Higher study graduates	8 (3.7)	74 (17.1)	82 (12.6)	
Marital status				
Unmarried	13 (0.6)	33 (7.6)	46 (7.1)	0.794*
Married	193 (88.5)	371 (85.7)	564 (86.6)	
Widowed	12 (5.5)	28 (6.5)	40 (6.1)	
Divorced	0 (0)	1 (0.2)	1 (0.2)	
Occupation				
Agriculture	73 (33.5)	32 (7.4)	105 (16.1)	< 0.001
Business	62 (28.4)	180 (41.6)	242 (37.2)	
Student	2 (0.9)	16 (3.7)	18 (2.8)	
Service	14 (6.4)	49 (11.3)	63 (9.7)	
Household work	56 (25.7)	117 (27)	173 (26.6)	
Retired	3 (1.4)	24 (5.5)	27 (4.1)	
Others	8 (3.7)	15 (3.5)	23 (3.5)	
Monthly income of family (Rs)				
< 20,000	113 (51.8)	102 (23.6)	215 (33.0)	< 0.001
20,000–40,000	54 (24.8)	171 (39.5)	225 (34.6)	
> 40,000	28 (12.8)	128 (29.6)	156 (24.0)	
Do not know	23 (10.6)	32 (7.4)	55 (8.4)	
Socio-economic status (SES)				
Quartile 1	103 (47.2)	26 (6.0)	129 (19.8)	0.665
Quartile 2	68 (31.2)	62 (14.3)	130 (20)	
Quartile 3	32 (14.7)	98 (22.6)	130 (20)	
Quartile 4	10 (4.6)	120 (27.7)	130 (20)	
Quartile 5	3 (1.4)	127 (29.7)	130 (20)	
Knowledge level				
High score	0	15 (3.5)	15 (2.3)	< 0.001
Low score	33 (15.1)	214 (49.4)	247 (37.9)	
No knowledge	185 (84.95)	204 (47.1)	389 (59.8)	
Attitude level				
High score	23 (65.7)	172 (75.4)	195 (74.1)	0.299
Low score	12 (34.3)	56 (24.6)	68 (25.9)	
Practice level				
High score	8 (22.9)	48 (21.0)	56 (21.2)	0.409*
Low score	27 (77.1)	181 (88.2)	208 (78.8)	

All *P*-values are based on chi-square analysis of numbers in highland and lowland groups except those indicated by an asterisk (*), which are based on Fisher’s exact test

### Knowledge and awareness about signs and symptoms of DF

Both the quantitative and qualitative data showed similar results. The HHS results revealed that 264 (40.6%) of the HHS participants had previously heard about DF, with significantly higher numbers in the lowland (86.7%) compared to the highland areas (*P* < 0.001). The majority of the participants were able to correctly identify general symptoms of DF, such as fever (91.7%) and headache (61.4%), respectively with a statistically significant difference between the highland and lowland areas (*P* < 0.05) (Additional file 2). However, for other typical symptoms of DF, such as joint pain *(P* = 0.010), muscle pain (*P* = 0.021), nausea/vomiting (*P* < 0.001), rash (*P* = 0.012), and diarrhea (*P* = 0.032), a significant higher number of participants from the lowland areas were able to correctly identify them (Additional file 2). Findings from the FGDs and IDIs also revealed that the majority of the lowland participants were aware about the common signs and symptoms like fever, joint pain, skin rashes and vomiting, while in the highland, only FCHVs and teachers were found to have knowledge on the signs and symptoms of DF. The FGD participants in lowland and highland summarized their perception and knowledge as follows:

*“Fever, joint pain, pain behind the eyes and the skin rashes are the symptoms of dengue, as I know*”, FGD- male participant, Chitwan district (Lowland).

*“I haven’t heard about it's (dengue) symptoms but I have heard that the dengue is transmitted to the people if the mosquitoes bite the human after biting the duck. Nevertheless, I don’t know about it’s effect and what happens to him or her if they suffer from it”,* FGD-male participant, Nuwakot district (Highland).

### Knowledge of dengue virus transmission and vector

The majority of the HHS participants (93.2%) knew that not all mosquitoes can transmit DENV, but only 12.1% knew that *Aedes* mosquitoes transmit DENV. Both responses were not significantly different between the highland and lowland study populations. Similarly, most of the HHS participants (84.1% and 84.8%, respectively) were aware that flies and ticks do not transmit DENV (Additional file 3). In the HHS, 64.6% of the lowland and 28.6% of the highland participants knew that DENV transmitting mosquitoes bite during daytime (*P* < 0.001) (Additional file 3). About 87% of all the HHS participants reported that the mosquitoes breed in standing water, although a significantly higher number were from lowland areas (*P* = 0.005) (Additional file 3). The IDI participants from both areas were aware of the *Aedes*, the vector and its day-biting behavior. However, the FGD participants of the lowland areas were only able to explain the vector. Participants in lowland and highland areas expressed their knowledge as follows:

*“I heard that the mosquitoes having white stripes on their bodies are the mosquitoes that transmit dengue. They have white stripes in their head too just like tigers”,* FGD- female participant, Chitwan district (Lowland).

*“We do not know about the diseases caused by the bite of mosquitoes. I even hear first time that the mosquitoes bite can cause diseases”,* FGD-female participant, Nuwakot district (Highland).

*“Mosquitoes with stripes, called as Aedes aegypti and Aedes albopictus are responsible to transmit DF. This type of mosquitoes’ bites people during the day time”,* IDI- Public health officer, Lalitpur district (Lowland).

### Sources of information on DF

**Fig. 2:**
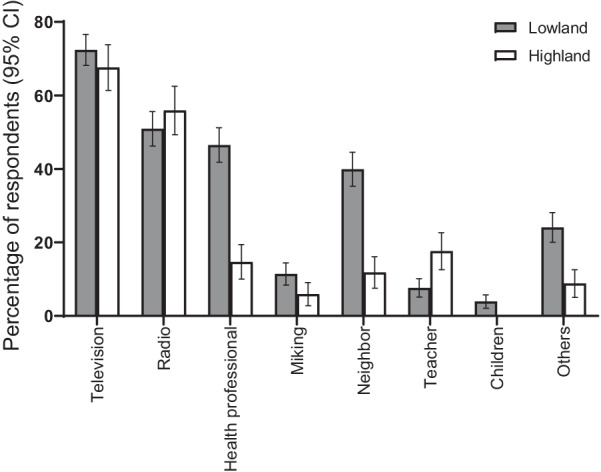
Sources of information on dengue fever. Error bars represent 95% confidence intervals

The majority of the HHS participants reported that they had received information about DF via television (71.2%), followed by radio (51.3%), but very few via teachers and children (Fig. 2). Only the sources of information like health professional, neighbor and other sources (newspaper and relatives), were statistically significant (*P* < 0.05) between the highland and lowland areas. The FGD participants also reported radio, television and neighbors as being main sources of information, as illustrated by the following quote:

*“I have heard about dengue from radio and television. One of our neighbor has also suffered from dengue for one month”,* FGD-male participant, Dhading district (Lowland).

### Attitudes and perceived risk towards dengue fever

Most of the HHS participants strongly agreed (63.3%) or agreed (29.2%) that DF is a serious illness (Additional file 4), while less than 50% strongly agreed that they were at risk of getting dengue. The attitude of people living in the highland and lowland areas were not statistically significantly different except for the statement that DF can be prevented (*P* < 0.001) (Additional file 4). The IDI participants of both highland and lowland areas reported the same view when queried on their perceived susceptibility towards the risk of future dengue epidemics. Most of them perceived that there is a higher chance of increasing dengue outbreaks, and being infected due to increasing temperature, as well as due to the movement of a dengue-infected person from endemic areas to non-endemic areas. The IDI participants in both areas summarized their perception as follows:

“*There is an increased risk of dengue outbreak in Kathmandu in the later days. The climate here is becoming hotter, the same as Terai region (tropical lowland). We can say that Kathmandu is at high risk as people from other districts are traveling here for different purposes”,* IDI-Kathmandu district (Lowland).

*“Of course, here is a risk of dengue outbreak in the coming days. If any dengue patient comes here from Chitwan (lowland) then, it can be transmitted to other people of here”,* IDI-Nuwakot district (Highland)*.*

### Prevention and control practices against DF

Various preventive measures were mentioned during the HHS, FGDs and IDIs. As revealed by the HHS, the commonly used preventive measures to reduce exposure to the mosquitoes were using nets in doors and windows (72.3%), eliminating standing water around the house (87.1%), cutting down bushes in the yard (86%), preventing water stagnation (88.3%), and using mosquito coils (63.6%) (Additional file 5). Responses on such preventive practices as using insecticides sprays, using nets in doors and windows, eliminating standing water around the house, preventing water stagnation, cleaning of garbage/trash and using fans were significantly more common among the lowland participants (*P* < 0.05) (Additional file 5). Similarly, the preventive measures mentioned by the FGD and IDI participants from the lowland areas, were the use of insecticides sprays, electric vaporing mats, mosquito coils, a mosquito net around the bed or installed in windows and doors, the use of coconut and mustard oil on the body, wearing long-sleeved clothes while going outside for work, cleaning the surroundings, managing stagnant water, etc. However, the IDIs from the highland areas revealed that the participants were not adopting any preventive or control measures due to there being only a few numbers of mosquitoes in their area. Here are the quotes from some of these participants:

*“We are not using anything until now to get rid of mosquitoes. We even do not use the mosquito net. We do not have to use anything because there are very few numbers of mosquitoes”,* IDI-female participant, Rasuwa district (Highland)*.*

*“We use bed nets, liquid (goodnight) and coil while sleeping to avoid the bites of mosquitoes”,* IDI- female participant, Dhading district (Lowland).

*“We use to cover our water containers because now we knew that the mosquitoes lay eggs in clean water. We also use to clean our surroundings and manage the stagnant water so that they cannot find places to lay eggs*”, FGD-male participant, Chitwan district (Lowland).

However, the IDI participants shared different experiences regarding the perception of the community towards DF preventive and control practices. They reported the lack of self-motivation in the community towards vector prevention and control. A male public health officer summarized his experienced as,

*“People think that cleaning their surroundings is not their work. They think it as the responsibility of the health-related persons or District Public Health Office to spray insecticides at their houses as well as their surroundings”,* IDI-Public health officer, Chitwan district (Lowland).

### Correlation between knowledge, attitude and practice

There were no statistically significant correlations between the knowledge, attitude and practice domains in both the highland and lowland communities (Table 3).

**Table 3:** Correlation between knowledge, attitude and practice scores

Variables	Correlation coefficient (r_s_) with 95% CI			*P*-value
	Highland	Lowland	Total	
Knowledge-attitude	0.51 (0.21–0.72)	0.48 (0.37–0.57)	0.49 (0.40–0.58)	0.496
Knowledge-practice	0.44 (0.13–0.67)	0.25 (0.12–0.37)	0.34 (0.22–0.44)	0.340
Attitude-practice	0.23 (0.10–0.53)	0.23 (0.11–0.35)	0.28 (0.16–0.38)	0.279

All *P*-values were obtained by Bootstrap method showing the correlation coefficient in highland and lowland groups

r_s_: Spearman rank correlation coefficients

CI: Confidence intervals

### Effect of socio-economic factors on the KAP level of DF and its prevention

Regarding the KAP scores, 2.3% of the participants achieved at least 80% (high score) on the knowledge score, 74.1% obtained at least 80% (high score) on the attitude score and 21.2% obtained at least 80% (high score) on the preventive practice score (Table 2). However, the KAP level between the highland and lowland dwellers were not statistically significantly different except for the knowledge level (*P* < 0.001). In the univariate analysis of the associations between knowledge and socio-economic variables of the study population (Table 4), we found decreasing odds of having low knowledge (OR: 0.17; *P* < 0.001) if the participants were inhabitant of lowland compared to participants of highland. Similarly, decreasing odds of having low knowledge was identified if the participants were literate, had secondary or higher secondary education level compared to participants who were illiterate (Table 4). Age, monthly income, SES and occupation were also significantly associated with low knowledge (*P* < 0.001; Table 4). After excluding insignificant predictor factors (*P* > 0.25) from the analysis, the multivariate model revealed that the area of residence, age and educational level were only independent predictor factor of knowledge regarding DF (Table 4).

**Table 4:** Univariate and multiple logistic regression analysis showing the predictors of knowledge level (Low knowledge vs no knowledge)

Independent variable	Univariate		Multivariate	
	OR (95% CI)	*P*-value	aOR (95% CI)	*P*-value
Area of residence				
Highland (R)	1	< 0.001	1	< 0.001
Lowland	0.17 (0.11–0.25)		0.29 (0.16–0.53)	
Gender				
Male (R)	1	0.828	–	
Female	1.03 (0.74–1.44)			
Age group (years)				
15–29 (R)	1	0.033	1	0.011
30–44	0.55 (0.34–0.87)		0.46 (0.25–0.84)	
45–59	0.64 (0.38–1.07)		0.31 (0.15–0.64)	
≥ 60	0.93 (0.52–1.68)		0.58 (0.23–1.49)	
Educational level				
Illiterate (R)	1	< 0.001	1	< 0.001
Literate	0.36 (0.20–0.66)		0.35 (0.16–0.78)	
Secondary	0.26 (0.13–0.49)		0.18 (0.08–0.43)	
Higher secondary	0.22 (0.12–0.41)		0.17 (0.07–0.44)	
High study graduates	0.12 (0.06–0.23)		0.08 (0.03–0.22)	
Monthly income (Rs)				
< 20,000 (R)	1	< 0.001	1	0.258
20,000–40,000	0.43 (0.29–0.65)		0.85 (0.50–1.42)	
> 40,000	0.50 (0.32–0.77)		1.28 (0.67–2.42)	
Do not know	1.06 (0.54–2.07)		1.79 (0.71–4.54)	
Socio-economic status (SES)				
Q1 quartile (R)	1	< 0.001	1	0.686
Q2 quartile	0.41 (0.22–0.74)		0.65 (0.32–1.35)	
Q3 quartile	0.27 (0.15–0.49)		0.82 (0.38–1.79)	
Q4 quartile	0.17 (0.10–0.31)		0.79 (0.34–1.82)	
Q5 quartile	0.21 (0.12–0.38)		1.02 (0.41–2.50)	
Ethnicity				
Dalit (R)	1	< 0.001	1	0.520
Disadvantaged Janajatis	2.45 (1.08–5.54)		0.79 (0.25–2.52)	
Disadvantaged non-Dalit Terai caste	0.94 (0.17–4.99)		0.76 (0.11–5.28)	
Religious minorities	0.70 (0.04–12.43)		0.19 (0.01–4.66)	
Relatively advantaged Janajatis	0.90 (0.40–2.01)		0.53 (0.17–1.65)	
Uppercaste	0.78 (0.35–1.70)		0.82 (0.27–2.51)	
Occupation				
Agriculture (R)	1	< 0.001	1	0.175
Business	0.38 (0.22–0.66)		0.84 (0.41–1.71)	
Student	0.11 (0.03–0.34)		0.17 (0.04–0.77)	
Service	0.29 (0.14–0.58)		1.11 (0.43–2.85)	
Household work	0.38 (0.21–0.67)		0.65 (0.31–1.37)	
Retired	0.24 (0.10–0.59)		0.53 (0.16–1.79)	
Others (teachers, carpenters)	0.75 (0.26–2.13)		1.04 (0.30–3.56)	
Affiliated to social insurance?				
Yes (R)	1	0.003	1	0.822
No	(1.33–2.91)		1.08 (0.65–1.78)	
Do not know	1.44 (0.31–6.69)		0.51 (0.03–7.44)	
Part of health care system as an affiliate or beneficiary?				
Yes (R)	1	0.006	1	0.002
No	3.71 (1.66–8.29)		5.80 (2.19–15.40)	
Do not know	4.44 (0.90–21.87)		6.65 (0.45–96.32)	

OR = Odd ratio, aOR = adjusted odds ratio, CI = confidence intervals, R: reference category

Due to very few high score events (15 in total), the logistic regression analysis was performed between low knowledge and no knowledge scores

In the univariate analysis (Table 5) of the associations between attitude and socio-demographic variables, we found increased odds of acquiring high attitude scores if the participants were literate compared to illiterate (OR: 3.20; 95% CI: 1.02–10.04). However, none of the socio-economic variables were associated with the attitude towards DF in multivariate analysis (Table 5). Similarly, we did not find any association between practice scores and socio-demographic variables (Table 6).

**Table 5:** Univariate and multiple logistic regression analysis showing predictors of attitude level (high vs. low) (n = 264)

Independent variable	Univariate		Multivariate	
	OR (95% CI)	*P*-value	aOR (95% CI)	*P*-value
Household location				
Highland (R)	1	0.224	1	0.74
Lowland	1.60 (0.74–3.42)		0.84 (0.32–2.25)	
Gender				
Male (R)	1	0.758	–	–
Female	0.91 (0.51–1.63)			
Age group (years)				
15–29 (R)	1	0.269	–	–
30–44	2.95 (0.83–10.40)			
45–59	2.98 (0.97–9.07)			
≥ 60	2.36 (0.72–7.76)			
Educational level				
Illiterate (R)	1	0.036	1	0.036
Literate	3.20 (1.02–10.04)		2.66 (0.68–10.33)	
Secondary	1.03 (0.46–2.30)		0.72 (0.28–1.82)	
Higher secondary	0.39 (0.13–1.14)		0.27 (0.08–0.85)	
High study graduates	1.10 (0.48–2.51)		0.89 (0.36–2.20)	
Monthly income (Rs)				
< 20,000 (R)	1	0.864	–	–
20,000–40,000	0.62 (0.18–2.10)			
> 40,000	0.74 (0.23–2.34)			
Do not know	0.64 (0.19–2.14)			
Socio-economic status				
Q1 quartile (R)	1	0.864	–	–
Q2 quartile	2.82(1.04–7.58)			
Q3 quartile	1.31 (0.55–3.11)			
Q4 quartile	0.79 (0.33–1.91)			
Q5 quartile	1.13 (0.51–2.48)			
Occupation				
Agriculture (R)	1	0.531	–	–
Business	1.33 (0.20–8.48)			
Student	0.81 (0.14–4.44)			
Service	0.75 (0.93–6.04)			
Household work	0.60 (0.09–3.87)			
Retired	1.15 (0.20–6.3)			
Others	0.19 (0.14–2.62)			
In the past years has anyone been infected with dengue at your home?				
No (R)	1	0.306		
Yes	1.93 (0.54–6.86)			
In the past years has anyone been infected with dengue in your neighbors?				
No (R)	1	0.151	1	0.094
Yes	1.69 (0.82–3.48)		2.12 (0.87–5.11)	

n = 264 (only those who have heard about dengue), OR = odds ratio, aOR = adjusted odds ratio, CI = confidence interval, R = reference category

**Table 6:** Univariate and multivariate logistic regression analysis showing the predictors of practice level (high vs. low) (n = 264)

Independent variable	Univariate		Multivariate	
	OR (95% CI)	*P*-value	aOR (95% CI)	*P*-value
Household location				
Highland (R)	1	0.376	–	–
Lowland	0.64 (0.24–1.69)			
Gender				
Male (R)	1	0.527	–	–
Female	1.29 (0.58–2.84)			
Age group (years)				
15–29 (R)	1	0.932	–	–
30–44	0.76 (0.18–2.87)			
45–59	1.03 (0.31–3.33)			
≥ 60	1.00 (0.27–3.59)			
Educational level				
Illiterate (R)	1	0.431	–	–
Literate	1.01 (0.18–5.49)			
Secondary	1.37 (0.43–4.34)			
Higher secondary	1.17 (0.33–4.12)			
High study graduates	0.56 (0.19–1.60)			
Monthly income (Rs)				
< 20,000 (R)	1	0.477	–	–
20,000–40,000	0.70 (0.14–3.54)			
> 40,000	1.26 (0.25–6.31)			
Do not know	1.40 (0.26–7.55)			
Socio-economic status				
Q1 quartile (R)	1	0.811	–	–
Q2 quartile	1.25 (0.31–4.99)			
Q3 quartile	1.19 (0.40–3.54)			
Q4 quartile	1.09 (0.40–2.99)			
Q5 quartile	1.93 (0.66–5.65)			
Occupation				
Agriculture (R)	1	0.758	–	–
Business	1.75 (0.13–22.77)			
Student	1.55 (0.16–14.20)			
Service	0.91 (0.06–12.32)			
Household work	0.69 (0.07–6.90)			
Retired	1.18 (0.12–11.01)			
Others	0.61 (0.52–7.24)			
Affiliated to social insurance?				
Yes (R)	1	0.588	–	–
No	3.50 (0.28–42.63)			
Part of health care system as an affiliate or beneficiary?				
Yes (R)	1	0.066	–	–
No	9.50 (0.68–131.99)			
Do not know	15.63 (1.37–178.18)			
In the past years has anyone been infected with dengue at your home?				
No (R)	1	0.787	–	–
Yes	0.81 (0.17–3.68)			
In the past years has anyone been infected with dengue in your neighbors?				
No (R)	1	0.110	1	0.136
Yes	1.90 (0.86–4.20)		1.84 (0.82–4.14)	

n = 264 (only those who have heard about dengue), OR = odds ratio, aOR = adjusted odds ratio, CI = confidence interval, R = the reference category

## Discussion

In the present study, both qualitative and quantitative findings provide similar information regarding the knowledge, attitude and practice of people residing in the highland and lowland areas of Central Nepal. According to our present study, only a small proportion of the HHS (2.3%) achieved a high knowledge score and high proportion achieved low (37.9%) and no (59.8%) knowledge score, while a large proportion (74.1%) achieved high attitude scores and a fairly-high proportion (21.2%) achieved high practice score regarding DF. Furthermore, both the qualitative (FGDs, IDIs) and quantitative (HHS) results indicate that people living in the lowland areas had better knowledge on DF than people living in highland areas, however no significant differences were found in the attitude and practice categories. Results from the HHS and FGDs illustrate, that while the majority of participants obtained high attitude scores towards DF, they had no adequate knowledge on DF and were not completely adopting the preventive practices in order to reduce the breeding sites of the dengue vectors. This lack in knowledge and practice behavior reveals the urgent need for massive dengue awareness campaigns in both areas. Moreover, providing health education about the disease should be compulsory to ensure that the Nepalese people can better understand DF, improve their knowledge on dengue transmission and on the preventive and control measures in order to reduce future dengue epidemics.

The qualitative data revealed that almost all of the IDI participants, except FGD participants in our study had heard and had knowledge of DF. However, only 40.6% of the HHS participants had previously heard about DF, with significantly more of these participants being from the lowland areas. This finding is consistent with a previous study conducted in Eastern Nepal [41]. However, in similar studies conducted in Australia, India and Pakistan, the majority of people (> 80%) had heard about DF [67–69] which is relatively higher than in Nepal. DF epidemics in those countries were reported much earlier, in the 1980 [12, 70], whereas in Nepal, the first DF outbreak was reported in 2006 [15], this may be the reason for why Nepalese people are less familiar with DF. Fever and headache were the most quoted symptoms in our study which are comparable with similar studies conducted in Sri Lanka, India, Yemen, Vientiane, Australia and Malaysia [67, 68, 71–73]. The major clinical features reported during the 2016 DF outbreak in Nepal were fever (100%), headache (71.3%), rashes (11.3%), retro-orbital pain (23.5%), vomiting (23.4%), joint pain (32.1%), and thrombocytopenia (85.7%), and minor symptoms were comprised abdominal pain and a feeling of restlessness [18]. However, most of the community people were unable to relate other common signs and symptoms of DF except for fever and headache, while the public health officers and FCHVs were aware of the other symptoms such as skin rashes, joint pain, eye pain and fatigue. The different perceptions and experiences of people with fever in relation to other illness, such as malaria, typhoid fever and seasonal flu may sometimes create confusion with the actual disease symptoms [74]. We found high knowledge level on the signs and symptoms of DF in the lowland community compared to the highland community; this may be due to the frequent DF outbreaks that have occurred in the lowland areas [12], while the participants in the highland neither personally experienced the disease nor witnessed a case from a close relative, friend or neighbor.

*Ae. aegypti* and *Ae. albopictus* are the most common vectors responsible for transmitting DF to humans [75]. Most of the HHS participants in our study thought that flies, ticks and all types of mosquitoes cannot transmit dengue but unfortunately, very few participants could state the *Aedes* mosquitoes as the major, responsible vector to transmit DF. A few lowland FGD participants were able to describe *Aedes,* but they could not name the vector. Conversely, the highland community, except teachers and health workers, did not even know that the mosquito’s bite could transmit dengue. The presence of fewer numbers of the mosquito vectors in the highland areas [20] might also be the plausible reason behind the ignorance of the highland dwellers towards mosquito vectors. In contrast to our study, a study from Malaysia showed that 97% of the participants knew that *Ae. aegypti* was the specific mosquito that causes DF [76]. Dengue was reported quite earlier in Malaysia (1901) than in Nepal, followed by more frequent outbreaks [77]. This may have influenced the population’s knowledge about the dengue vector and caused this to be handled down the generations in Malaysia than in Nepal. Regarding the feeding behavior of the dengue vectors, the dengue vector’s bite occurs mostly after sunshine and before sunset [78]. Meanwhile, more than half of the HHS participants as well as public health professionals, health workers, teachers and FCHVs, were aware about the daytime biting habit of the dengue vectors. This finding is comparable with other studies carried out in Thailand [64] and Sri Lanka [71]. There is also increasing evidence of DENV transmission by blood and organ transplantation [79–82], suggesting the increasing threat to blood supplies, especially in endemic regions. Interestingly, 70.5% of the HHS participants in our study stated that DF could be transmitted by blood transfusion, thus the negative image associated with blood transfusion as a risk of transmitting diseases in general might influence people’s perceptions [83]. Hence, the provision of adequate and relevant information should be made readily available to all layers of the communities [84]. However, most participants in our study mentioned radio and television as their main sources of information regarding DF, followed by health professionals and neighbors. Similar findings were reported from Jamaica, India, Vientiane and Malaysia [68, 76, 85, 86]. Surprisingly, very few participants had received DF information through teachers and children, indicating the lack of an updated health education in the educational institutions. Thus, the development of school-based educational programs is very important in order to enhance the knowledge of DF for both teachers and students; this could motivate all actors towards dengue prevention and control. Furthermore, school-based dengue prevention and control programs could provide a sustainable practice for community awareness through the involvement of children. Thus, this could help to prevent the disease spread in endemic areas, and control future dengue epidemics [87] in non-dengue endemic regions. Health professionals compared to highland areas better informed participants from lowland areas about dengue. The difficult geographical landscape of the highland regions, linked with rough and poor maintained roads [88], presumably reduces the mobilization of health workers in the highland areas. Meanwhile, the higher frequency of DF outbreaks in the lowlands may lead the health professionals to talk more about DF in the lowland areas than in the highland areas. Thus, this suggests that the health authorities of each area could customize their channels of information by emphasizing the collaboration between communities, community leaders, FCHVs, local political leaders including religious bodies, local non-governmental organizations, local youth clubs and local educational institutions. This could help to establish a cordial relationship between the communities and the stakeholders, including health professionals, which would also help to enhance the knowledge of DF among different communities and motivate themselves to improve their DF preventive and control practices.

The high attitude scores acquired by the majority of the HHS participants in our study (74.1%) showed a greater concern of the people towards DF; similar findings, i.e., high attitude, were reported in Pakistan [69, 89] and Yemen [90]. The majority of the people perceived DF as a serious disease and seemed supportive towards dengue prevention and control. However, most of them did not consider themselves to be at risk of having DENV infection; we believe that, these participants did not have previous communication with dengue-infected persons in their neighborhood or that, in the community, there had scarcely been any reports of severe dengue infection. Nonetheless, our qualitative findings revealed that the people from both the highland and lowland areas working in the health management sectors believed that the lowland and highland areas are at risk of increasing dengue outbreaks due to increasing temperature and mobility of people from dengue-endemic regions to non-endemic regions. Recent DF outbreaks, with reported cases from the lowlands to the middle and high, mountainous regions of Nepal, are clear evidence of the growing threat of DF epidemics with the changing climate [17]. Discarded tires, mud pots, ditches, plastic buckets, cement tanks, tree holes, rocks and other plastic containers in indoor and outdoor locations have been investigated as major breeding sites for *Aedes* mosquitoes as described in various studies [91–94]. In addition, the control of the dengue vectors has mainly been approached by source reduction, such as the elimination and management of water-holding containers and discarded tires [95, 96]. The most promising thing is that more than 80% of the participants from both the highland and lowland areas were positive towards the fact that stagnant water in discarded tires, broken pots and bottles are the breeding sites of those mosquitoes and are aware that controlling the breeding places of mosquitoes is a good strategy to prevent dengue. This finding is consistent with a similar study conducted in Nepal between 2011 and 2012 [40].

More people in our study were found to obtain high practice score than overall knowledge score. Similar findings were reported in studies from Sri Lanka [97], Vietnam [98, 99] and Malaysia [100] but contrary to other studies of Philippines [101] and Jamaica [85], which reported high levels of knowledge but low level of practices. Despite having a poor knowledge of the symptoms and transmission modes regarding DF, most of the highland and lowland dwellers (< 70%) had a very good knowledge on preventive measures. This might be because the questions asked at the practice level were linked with the people’s daily practices in order to control other mosquitoes and were not DF-specific [40]. The translation of this knowledge regarding preventive practices was partially observed in our study. HHS participants stated that they were covering water containers in the home, focusing on cleaning of garbage/trash, cutting down bushes in the yard, turning containers upside down, and preventing water stagnation in order to eliminate mosquito-breeding sites and to reduce the mosquitoes. The other common practices mentioned by the HHS, FGDs and IDIs were the use of nets in doors and windows, fans, mosquito coils and covering the body with clothes. These preventive practices were reported less frequently in the highland areas as the people there reported a lower presence of mosquitoes. Nevertheless, it is a little discouraging that only a small proportion of the lowland participants considered the use of mosquito-repellent cream, insecticides sprays and professional pest control as methods of protection. Overall, only 21% participants of our study reported a high practices score; this is consistent with the studies in Jamaica [85] and Indonesia [62]. This may have been attributed by the people’s attitudes, where most of the participants in our study did not consider themselves to be at risk of DF. Besides, it is difficult to change a person’s behavior deeply embedded in structural factors and social determination [102], such as sleeping outdoors due to load shedding, affordability and the lack of resources including professional pest control, mosquito coils, mosquito repellent cream, etc.

In contrast to the study of Dhimal et al. [40], our study found a statistically significantly difference between the knowledge level of the highland and lowland dwellers. However, we could not observe any statistically significant difference in their attitudes and practice levels. Among the socio-demographic variables, the overall knowledge of the participants was associated with the area of residence (highland/lowland), age, educational level, monthly income, SES and occupation. People from the lowland areas if compared to the highland areas were less likely to have a low knowledge score. Frequent DF outbreaks and ongoing DF control programs during outbreak period might tend the people of lowland areas to gain at least some knowledge on DF. Similarly, participants with secondary or higher secondary education level were less likely to achieve low knowledge score on DF if compared to illiterate people. However, there was no significant association of the knowledge level with gender consistent with a study conducted in Indonesia [62]. Similarly, no significant association was found between preventive practices and socio-demographic characteristics including education level; this is consistent with a study conducted in Jamaica [85]. This implies that those demographic variables do not have significant bearings nor influence on how they behave to prevent dengue infection. One possible cause might be the lack of health education in the curricula regarding DF in each academic level [40]. Furthermore, the lack of school-based awareness programs regarding DF preventive and control measures might be the reason why the educational qualification of study’s participants did not contribute to the adoption of better preventive practices. Attitude was found to be positively associated with the educational level, a finding consistent with the results from Malaysia [76]. Literate participants were found to obtain high attitude scores if compared to illiterate people, indicating the role of education for changing the attitudes of people [40].

Our study shows a complete lack of high knowledge scores, and more participants with no knowledge scores in the highland areas regarding DF. However, in the lowland, at least 3.5% of the researched participants had acquired high knowledge scores on the signs, and symptoms and transmission of DF as well as the effective preventive and control measures against DF. Due to occurrence of frequent DF outbreaks and the DF burden in the lowlands of Nepal in recent years [17, 26, 35–37, 90] and accordingly, due to the awareness programs conducted in lowland during the outbreak periods might tend people of lowland to gain more knowledge on DF if compared to highland. Although the study of Shrestha et al. [106] reported the implementation of effective vector control strategies as a definite reason beside the reduction of dengue cases in Nepal up to 2016, the low level of DF-related knowledge and practices in this study raise a big question regarding the adoption of effective health educational programs in the communities. In the meantime, the huge dengue outbreak in 2019 [17] indicates that the ongoing dengue prevention and control programs in Nepal are not sufficient and need to be improve in order to prevent the increasing DF incidence in Nepal.

### Limitations and strengths

The findings of our study must be interpreted with caution regarding certain aspects. Being a cross-sectional survey, this study only evaluated the relationship based on one point and could not account for the dynamics of the relationships between the variables analyzed. Besides, it is also possible that some participants may have provided socially desirable responses to some questions, which may, thus, not have reflected their actual attitudes and practices [40, 85]. More importantly, the data was collected in densely populated urban and semi-urban areas of each altitudinal region, i.e., clustering of households in a 50 m radius around selection data collection site may not be representative for the districts and the whole country. Furthermore, the study area was categorized as lowland and highland by keeping the baseline at 1500 m amsl. Due to this criterion, four districts in the lowland and two districts in the highland were considered as sampling sites. Additionally, due to very low high score events (15 in total), we could not apply the logistic regression model to this variable; thereby this variable is excluded from the analysis (Table 4). A strength of the present study was its mixed-method design, which offered the opportunity to triangulate the findings in order to gain a deep understanding on the people’s KAP on DF. In order to compare our findings with a previously conducted study in Nepal, we adopted a similar methodology without any major modifications.

## Conclusion

The Nepalese people have a very high attitude level regarding DF. However, their knowledge and awareness sensitivity on DF and preventive practices regarding vector control remains at low level in both highland (> 1500 m amsl) and lowland (< 1500 m amsl) areas. Compared to people in the highland, the lowland people have more knowledge on DF but this knowledge is not adequate to prevent and control future dengue epidemics. For the effective prevention of future dengue epidemics, it is recommended to broaden the use and scope of mass media such as radio and television, to share DF information on a timely basis and with content that, potentially, could lead to behavioral changes in the people. The health authorities should highly customize their channels of information by emphasizing the collaboration required between communities and various stakeholders. However, the development of sufficiently and easily understandable IEC/BCC materials on DF is most important in order to bring awareness to the community people having different educational levels. Most importantly, the inclusion of health education in school and university curricula, as well as school-based preventive programs regarding DF is highly recommended to establish a sustainable chain of awareness especially in highland areas that lack quality health services as well as adequate health education.

## Supplementary Information

**Additional file 1:** Thresholds and methods used for scoring people’s knowledge, attitude and practice (KAP).

**Additional file 2:** Participant’s knowledge on signs and symptoms of dengue fever.

**Additional file 3:** Participants knowledge on dengue virus transmission and vector.

**Additional file 4:** Participants’ attitudes towards dengue fever.

**Additional file 5:** Participants’ preventive and control measures against dengue fever.

**Additional file 6:** Survey Questionnaire.

## Data Availability

The datasets used and/or analyzed during the current study are available from the corresponding author on reasonable request.

## Abbreviations

Amsl: Above mean sea level
aOR: Adjusted odds ratio
BCC: Behavioral change communication
CI: Confident interval
DENV: Dengue virus
DF: Dengue fever
DNN: Do not know
FCHV: Female community health volunteer
FGD: Focus group discussion
HHS: Household survey
IDI: In-depth Interview
IEC: Information education communication
KAP: Knowledge, attitude, and practice
OR: Odds ratio
SES: Socio-economic status
