# Supplementary material for: The knowledge, attitude and practice of community people on dengue fever in Central Nepal: a cross-sectional study
Source: BMC Infect Dis. 2022 May 12;22:454. doi: 10.1186/s12879-022-07404-4 (PMC9096776; doi:10.1186/s12879-022-07404-4)
Supplement: Supplementary file 1 — Additional file 1: Thresholds and methods used for scoring people’s knowledge, attitude and practice (KAP). [file 12879_2022_7404_MOESM1_ESM.docx]

**Additional file 1: Thresholds and methods used for scoring people’s knowledge, attitude and practice (KAP)**

| **KAP domain** | **Total questions/**  **scores** | **Scoring method** | **Criteria (80% cut off score)** | **Round off scores** | **Category** |
| --- | --- | --- | --- | --- | --- |
| Knowledge domain | 24 | Correct answer=1 | 19.2 | ≥19 | High score |
|  |  | Wrong / Do not know =0 |  | 1 to 18 | low score |
|  |  | Who have not heard about DF=0 for all responses |  | 0 | No knowledge |
| Attitude domain | 6 | Correct answer=1 | 4.8 | ≥5 | High score |
|  |  | Wrong answer/ do not know=0 |  | <5 | low score |
| Practice domain | 21 | Correct answer=1 | 16.8 | ≥17 | High score |
|  |  | Wrong answer/ do not know=0 |  | <17 | low score |

Note: People who had not heard about dengue prior to survey were scored as zero for all responses of questions used for knowledge domain
