# Supplementary material for: The knowledge, attitude and practice of community people on dengue fever in Central Nepal: a cross-sectional study
Source: BMC Infect Dis. 2022 May 12;22:454. doi: 10.1186/s12879-022-07404-4 (PMC9096776; doi:10.1186/s12879-022-07404-4)
Supplement: Supplementary file 2 — Additional file 2: Participant’s knowledge on signs and symptoms of dengue fever. [file 12879_2022_7404_MOESM2_ESM.docx]

**Additional file 2: Participant’s knowledge on signs and symptoms of dengue fever**

| **Variables** | **Highland**  **n (%)** | **Lowland**  **n (%)** | **Total**  **n (%)** | ***P*-value** |
| --- | --- | --- | --- | --- |
| Fever is a symptom of DF | | | | 0.004* |
| **Yes** | 27 (77.1) | 215 (93.9) | 242 (91.7) |  |
| No | 8 (22.9) | 14 (6.1) | 22 (8.3) |  |
| Headache is a symptom of DF | | | | 0.002 |
| **Yes** | 13 (37.1) | 149 (65.1) | 162 (61.4) |  |
| No | 22 (62.9) | 80 (34.9) | 102 (38.6) |  |
| Joint pain is a symptom of DF | | | | 0.010 |
| **Yes** | 5 (14.3) | 83 (36.2) | 88 (33.3) |  |
| No | 30 (85.7) | 146(63.8) | 176 (66.7) |  |
| Muscle pain is a symptom of DF | | | | 0.021 |
| **Yes** | 5 (14.3) | 77 (33.6) | 82 (31.1) |  |
| No | 30 (85.7) | 152 (66.4) | 182 (68.9) |  |
| Pain behind the eyes is a symptom of DF | | | | 0.060* |
| **Yes** | 1 (2.9) | 33 (14.4) | 34 (12.9) |  |
| No | 34 (97.1) | 196 (85.6) | 230 (87.1) |  |
| Nausea/vomiting are symptoms of DF | | | | <0.001 |
| **Yes** | 5 (14.3) | 109 (47.6) | 114 (43.2) |  |
| No | 30 (85.7) | 120 (52.4) | 150 (56.8) |  |
| Rash is a symptom of DF | | | | 0.012 |
| **Yes** | 1 (2.9) | 47 (20.5) | 48 (18.2) |  |
| No | 34 (97.1) | 182 (79.5) | 216 (81.8) |  |
| Diarrhea is common in DF | | | | 0.032* |
| **Yes** | 0 (0) | 26 (11.4) | 26 (9.8) |  |
| No | 35(100) | 203(88.6) | 238 (90.2) |  |
| Back pain is common in DF | | | | 0.087* |
| **Yes** | 0 (0) | 21 (9.2) | 21 (8) |  |
| No | 35 (100) | 208 (90.8) | 243(92) |  |
| Stomach pain is common in DF | | | | 0.143* |
| **Yes** | 0 (0) | 18 (7.9) | 18 (6.8) |  |
| No | 35 (100) | 211 (92.1) | 246 (93.2) |  |

All *P*-values are based on chi-square analysis of numbers in highland and lowland groups except those indicated by an asterisk (*), which are based on Fisher’s exact test.

Note: Correct answers are those with bold **responses**
