# Supplementary material for: The knowledge, attitude and practice of community people on dengue fever in Central Nepal: a cross-sectional study
Source: BMC Infect Dis. 2022 May 12;22:454. doi: 10.1186/s12879-022-07404-4 (PMC9096776; doi:10.1186/s12879-022-07404-4)
Supplement: Supplementary file 3 — Additional file 3: Participants knowledge on dengue virus transmission and vector. [file 12879_2022_7404_MOESM3_ESM.docx]

**Additional file 3: Participants knowledge on dengue virus transmission and vector**

| **Variables** | **Highland**  **n (%)** | **Lowland**  **n (%)** | **Total**  **n (%)** | **P-Value** |
| --- | --- | --- | --- | --- |
| All mosquitoes transmit dengue | | | | 0.072* |
| Yes | 5 (14.3) | 13 (5.7) | 18 (6.8) |  |
| **No** | 30 (85.7) | 216 (94.3) | 246 (93.2) |  |
| *Aedes* mosquitoes transmit dengue | | | | 0.780* |
| **Yes** | 3 (8.6) | 29 (12.7) | 32 (12.1) |  |
| No | 32 (91.4) | 200 (87.3) | 232 (87.9) |  |
| Flies transmit DF | | | | 0.778 |
| Yes | 5 (14.3) | 37 (16.2) | 42 (15.9) |  |
| **No** | 30 (85.7) | 192 (83.8) | 222 (84.1) |  |
| Ticks transmit DF | | | | 0.878 |
| Yes | 5 (14.3) | 35 (15.3) | 40 (15.2) |  |
| **No** | 30 (85.7) | 194 (84.7) | 224 (84.8) |  |
| Ordinary person to person contact transmit DF | | | | 0.260 |
| Yes | 11 (31.4) | 52 (22.7) | 63 (23.9) |  |
| **No** | 24 (68.6) | 177 (77.3) | 201 (76.1) |  |
| DF transmitted through food and water | | |  | 0.113 |
| Yes | 6 (17.1) | 69 (30.1) | 75 (28.4) |  |
| **No** | 29 (82.9) | 160 (69.9) | 189 (71.6) |  |
| DF can be transmitted by blood transfusion | | | | 0.146 |
| **Yes** | 21 (60) | 165 (72.1) | 186 (70.5) |  |
| No | 14 (40) | 64 (27.9) | 78 (29.5) |  |
| Knowledge about biting time of dengue mosquitoes | | | | <0.001 |
| **Correct knowledge (Day time)** | 10 (28.6) | 148 (64.6) | 158 (59.8) |  |
| Incorrect knowledge | 25 (71.4) | 81 (35.4) | 106 (40.2) |  |
| Mosquitoes breed in standing water | | | | 0.005* |
| **Yes** | 25 (71.4) | 206 (90) | 231 (87.5) |  |
| No | 10 (28.6) | 23 (10) | 33 (12.5) |  |
| Window screens and bed net reduce mosquitoes | | | | 0.760* |
| **Yes** | 31 (88.6) | 207 (90.4) | 238 (90.2) |  |
| No | 4 (11.4) | 22 (9.6) | 26 (9.8) |  |
| Insecticide sprays reduce mosquitoes and prevent Dengue | | | | 0.229 |
| **Yes** | 29 (82.9) | 168 (73.4) | 197 (74.6) |  |
| No | 6 (17.1) | 61 (26.6) | 67 (25.4) |  |
| Tightly covering water containers reduces mosquitoes | | | | 0.984 |
| **Yes** | 27 (77.1) | 177 (77.3) | 204 (77.3) |  |
| No | 8 (22.9) | 52 (22.7) | 60 (22.7) |  |
| Removal of standing water can prevent mosquito breeding | | | | 0.310 |
| **Yes** | 28 (80) | 198 (86.5) | 226 (85.6) |  |
| No | 7 (20) | 31 (13.5) | 38 (14.4) |  |
| Mosquito repellents prevent mosquitoes | | | | 0.943 |
| **Yes** | 28 (80) | 182 (79.5) | 210 (79.5) |  |
| No | 7 (20) | 47 (20.5) | 54 (20.5) |  |

All *P*-values are based on chi-square analysis of numbers in highland and lowland groups except those indicated by an asterisk (*), which are based on Fisher’s exact test.

Note: Correct answers are those with **bold responses**
