# Supplementary material for: The knowledge, attitude and practice of community people on dengue fever in Central Nepal: a cross-sectional study
Source: BMC Infect Dis. 2022 May 12;22:454. doi: 10.1186/s12879-022-07404-4 (PMC9096776; doi:10.1186/s12879-022-07404-4)
Supplement: Supplementary file 4 — Additional file 4: Participants’ attitudes towards dengue fever. [file 12879_2022_7404_MOESM4_ESM.docx]

**Additional file 4: Participants’ attitudes towards dengue fever**

| **Variables** | **Highland**  **n (%)** | **Lowland**  **n (%)** | **Total**  **n (%)** | **P-Value** |
| --- | --- | --- | --- | --- |
| Is DF a serious illness? | | | | 0.592* |
| **Strongly agree** | 21(60) | 146(63.8) | 167(63.3) |  |
| **Agree** | 10(28.6) | 67(29.3) | 77(29.2) |  |
| Disagree | 1(2.9) | 7(3.1) | 8(3) |  |
| Not sure | 3(8.6) | 9(3.9) | 12(4.5) |  |
| Are you at risk of getting dengue? | | | | 0.102* |
| **Strongly agree** | 10(28.6) | 56(24.5) | 66(25) |  |
| **Agree** | 4(11.4) | 54(23.6) | 58(22) |  |
| Disagree | 8(22.9) | 72(31.4) | 80(30.3) |  |
| Strongly disagree | 6(17.1) | 28(12.2) | 34(12.9) |  |
| Not sure | 7(20) | 19(8.3) | 26(9.8) |  |
| Can DF be prevented? | | | | <0.001* |
| **Strongly agree** | 17(48.6) | 127(55.7) | 144(54.8) |  |
| **Agree** | 6(17.1) | 80(35.1) | 86(32.7) |  |
| Disagree | 3(8.6) | 0(0) | 3(1.1) |  |
| Strongly disagree | 0(0) | 2(0.9) | 2(0.8) |  |
| Not sure | 9(25.7) | 19(8.3) | 28(10.6) |  |
| Controlling breeding places of mosquitoes is good strategy to prevent dengue | | | | 0.060* |
| **Strongly agree** | 21(60) | 131(57.2) | 152(57.6) |  |
| **Agree** | 6(17.1) | 68(29.7) | 74(28) |  |
| Disagree | 0(0) | 9(3.9) | 9(3.4) |  |
| Strongly disagree | 1(2.9) | 1(0.4) | 2(0.8) |  |
| Not sure | 7(20) | 20(8.7) | 27(10.2) |  |
| Stagnant water in discarded tires, broken pots and bottles are breeding places | | | | 0.098* |
| **Strongly agree** | 22(62.9) | 136(59.4) | 158(59.8) |  |
| **Agree** | 5(14.3) | 66(28.8) | 71(26.9) |  |
| Disagree | 1(2.9) | 3(1.3) | 4(1.5) |  |
| Not sure | 7(20) | 24(10.5) | 31(11.7) |  |
| Communities should actively participate in controlling the vectors of Dengue | | | | 0.081* |
| **Strongly agree** | 22(62.9) | 160(69.9) | 182(68.9) |  |
| **Agree** | 4(11.4) | 43(18.8) | 47(17.8) |  |
| Disagree | 0(0) | 4(1.7) | 4(1.5) |  |
| Strongly disagree | 0(0) | 1(0.4) | 1(0.4) |  |
| Not sure | 9(25.7) | 21(9.2) | 30(11.4) |  |

All *P*-values are based on chi-square analysis of numbers in highland and lowland groups except those indicated by an asterisk (*), which are based on Fisher’s exact test.

Note: Correct answers are those with **bold** **responses** (strongly agree or agree)
