# Supplementary material for: The knowledge, attitude and practice of community people on dengue fever in Central Nepal: a cross-sectional study
Source: BMC Infect Dis. 2022 May 12;22:454. doi: 10.1186/s12879-022-07404-4 (PMC9096776; doi:10.1186/s12879-022-07404-4)
Supplement: Supplementary file 5 — Additional file 5: Participants’ preventive and control measures against dengue fever. [file 12879_2022_7404_MOESM5_ESM.docx]

Additional file 5: Participants’ preventive and control measures against dengue fever

| **Variables** | **Highland**  **n (%)** | **Lowland**  **n (%)** | **Total**  **n (%)** | **P-Value** |
| --- | --- | --- | --- | --- |
| Prevent mosquito-man contact | | | | 0.062 |
| **Yes** | 6 (17.1) | 75 (32.8) | 81 (30.7) |  |
| No | 29 (82.9) | 154 (67.2) | 183 (69.3) |  |
| Use insecticide sprays to reduce mosquito numbers | | | | 0.024 |
| **Yes** | 7 (20) | 91 (39.7) | 98 (37.1) |  |
| No | 28 (80) | 138 (60.3) | 166 (62.9) |  |
| Use professional pest control to reduce mosquito numbers | | |  | 0.118 |
| **Yes** | 7 (20) | 76 (33.2) | 83 (31.4) |  |
| No | 28 (80) | 153 (66.8) | 181 (68.6) |  |
| Use nets in doors and windows to control mosquito numbers | | | | 0.001 |
| **Yes** | 17 (48.6) | 174 (76) | 191 (72.3) |  |
| No | 18 (51.4) | 55 (24) | 73 (27.7) |  |
| Eliminate standing water around the house to reduce mosquito numbers | | | | <0.001* |
| **Yes** | 21 (60) | 209 (91.3) | 230 (87.1) |  |
| No | 14 (40) | 20 (8.7) | 34 (12.9) |  |
| Cut down bushes in the yard to reduce mosquitoes | | | | 0.016* |
| **Yes** | 25 (71.4) | 202 (88.2) | 227 (86) |  |
| No | 10 (28.6) | 27 (11.8) | 37 (14) |  |
| Prevent water stagnation | |  |  | 0.003* |
| **Yes** | 25 (71.4) | 208 (90.8) | 233 (88.3) |  |
| No | 10 (28.6) | 21 (9.2) | 31 (11.7) |  |
| Use mosquito eating fish to reduce mosquito numbers | | | | 0.274* |
| **Yes** | 2 (5.7) | 31 (13.5) | 33 (12.5) |  |
| No | 33 (94.3) | 198 (86.5) | 231 (87.5) |  |
| Use mosquito coils to reduce mosquito numbers | | | | 0.631 |
| **Yes** | 21 (60) | 147 (64.2) | 168 (63.6) |  |
| No | 14 (40) | 82 (35.8) | 96 (36.4) |  |
| Cleaning of garbage/trash to reduce mosquito breeding opportunities | | | | 0.001* |
| **Yes** | 28 (80) | 221 (96.5) | 249 (94.3) |  |
| No | 7 (20) | 8 (3.5) | 15 (5.7) |  |
| Dispose water holding containers to reduce mosquito breeding opportunities | | | | 0.050 |
| **Yes** | 23 (65.7) | 184 (80.3) | 207 (78.4) |  |
| No | 12 (34.3) | 45 (19.7) | 57 (21.6) |  |
| Use mosquito repellent/ cream | | | | 0.447 |
| **Yes** | 12 (34.3) | 94 (41) | 106 (40.2) |  |
| No | 23 (65.7) | 135 (59) | 158 (59.8) |  |
| Use of fan |  |  |  | <0.001 |
| **Yes** | 5 (14.3) | 176 (76.9) | 181 (68.6) |  |
| No | 30 (85.7) | 53 (23.1) | 83 (31.4) |  |
| Use of smoke to drive away mosquitoes | | | | 0.199 |
| **Yes** | 15 (42.9) | 73 (31.9) | 88 (33.3) |  |
| No | 20 (57.1) | 156 (68.1) | 176 (66.7) |  |
| Covering body with clothes to protect against mosquito bites | | | | 0.356 |
| **Yes** | 19 (54.3) | 143 (62.4) | 162 (61.4) |  |
| No | 16 (45.7) | 86 (37.6) | 102 (38.6) |  |
| Do nothing to reduce mosquitoes numbers or mosquito breeding sites | | | | 1.000* |
| Yes | 2 (5.7) | 19 (8.3) | 21(8) |  |
| **No** | 33 (94.3) | 210 (91.7) | 243 (92) |  |
| Eliminate mosquito breeding sites | | | | 0.010 |
| **Yes** | 18 (51.4) | 167 (72.9) | 185 (70.1) |  |
| No | 17 (48.6) | 62 (27.1) | 79 (29.9) |  |
| Cover water containers in the home to prevent mosquito breeding | | | | 0.103* |
| **Yes** | 27 (77.1) | 202 (88.2) | 229 (86.7) |  |
| No | 8 (22.9) | 27 (11.8) | 35 (13.3) |  |
| Frequently cleaning water filled containers and ditches around the house | | | | 0.973* |
| **Always** | 20 (57.1) | 126 (55) | 146 (55.3) |  |
| **Often** | 11 (31.4) | 66 (28.8) | 77 (29.2) |  |
| Sometimes | 4 (11.4) | 27 (11.8) | 31 (11.7) |  |
| Never | 0 (0) | 3 (1.3) | 3 (1.1) |  |
| Don’t know | 0 (0) | 7 (3.1) | 7 (2.7) |  |
| Government sprays insecticides for mosquito control | | | | 0.484* |
| Yes | 1( 2.9) | 18 (7.9) | 19 (7.2) |  |
| No | 34 (97.1) | 211 (92.1) | 245 (92.8) |  |
| Turning containers upside down to avoid water collection | | | | 0.951 |
| **Yes** | 26 (74.3) | 169 (73.8) | 195 (73.9) |  |
| No | 9 (25.7) | 60 (26.2) | 69 (26.1) |  |

All *P*-values are based on chi-square analysis of numbers in highland and lowland groups except those indicated by an asterisk (*), which are based on Fisher’s exact test.

Note: Correct answers are those with **bold responses**.
