# Supplementary material for: The knowledge, attitude and practice of community people on dengue fever in Central Nepal: a cross-sectional study
Source: BMC Infect Dis. 2022 May 12;22:454. doi: 10.1186/s12879-022-07404-4 (PMC9096776; doi:10.1186/s12879-022-07404-4)
Supplement: Supplementary file 6 — Additional file 6: Survey Questionnaire. [file 12879_2022_7404_MOESM6_ESM.docx]

|  |  |  |  |
| --- | --- | --- | --- |

**Survey Questionnaire**

**Identification number**

**Eco-bio-social drivers for effective *Aedes* vector prevention & control along a climatic gradient in Nepal - NAECO**

**A SURVEY INFORMATION AND GEO-REFERENCING**

| **SN** | **Variables** |  |
| --- | --- | --- |
| A1 | Name of participant |  |
| A2 | Date of interview | Day  Month Year |
| A3 | Municipality |  |
| A4 | Cluster |  |
| A5 | Address |  |
| A6 | Latitude |  |
| A7 | Longitude |  |
| A8 | Height | ====================== |

**B= RESEARCH PARTICPANT INFORMATION**

| **S.N** | **Variables** |
| --- | --- |
| B1 | Full name==================================================================================== |
| B2 | In what district and gaupalika/municipality were you born?  a= District ========================= b= Rural municipality==============  c. Municipality===========================  d= Other country====================== |
| B3 | Age=============== in years |
| B4 | Sex Male Female |
| B5 | Which of the following ethnic groups are you belong to?  1 = Dalit 2 = Disadvantaged Janajatis 3 = Disadvantaged non Dalit Terai caste groups 4 = Religious minorities  5 = Relatively advantaged Janajatis 6 = Upper caste |
| B6 | Educational qualification:  1. Illiterate 2. Literate (Informal education) 3. Secondary  4. Higher secondary 5. Higher study graduates |
| B7 | Marital status: 1. Unmarried 2. Married 3.Widow 4. Divorced |
| B8 | Occupation :  1. Agriculture 2. Business 3. Student 4.Service  5. Household work 6. Retired 7. Others…………….. |
| B9 | Are you part of the healthcare system as an affiliate or beneficiary?  1 = Yes 2 = No 3 = do not know |
| B10 | Are you affiliated to social insurance?  1 = Yes 2= No 3= Do not know |
| B11 | What is the monthly family income? (DO NOT READ OPTIONS, WAIT FOR ANSWERS)  1 = less than NRs. 10000 2 = NRS. 10,000-20,000  3 = 20,000-30,000 4 = 30,000-40,000  5 = 40,000-50,000 6 = more than 50,000  7 = do not know |

**C= CHARACTERISTICS OF THE HOUSING AND BASIC UTILITIES**

| **S.N** | **Variables** | | |
| --- | --- | --- | --- |
| C1 | What type of housing is this?  1 = House  2 = Apartment  3 = Room(s) in tenancies  4 = Room(s) in other type of structure  5 = Other type of housing (tent, carp, wagon, ship, natural refuge, bridge, etc.) | | |
| C2 | How many floor are in your house?  If more than one, in which of them do you live? ……… | | |
| C3 | OBSERVE AND WRITE DOWN THE PREDOMINANT MATERIAL OF THE FLOOR OF THE HOUSE OCCUPIED BY THE HOME  1 = Marble, parquet, polished and lacquered wood;  2 = Carpet, wall to wall carpet;  3 = Tile, ceramic tile, vinyl, tablet, brick, polished non-lacquered wood;  4 = Rough wood, wood board or plank, other vegetal;  5 = Cement, gravel;  6 = Soil/sand | | |
| C4 | Which of these spaces does your house have? | | |
|  | 1 = Courtyard 1 = Yes; 2 = No |  |  |
|  | 2 = Lot or solar 1 = Yes; 2 = No |  |  |
|  | 3 = Front yard 1 = Yes, 2 = No  4 = Garage or parking spot 1 = Yes; 2 = No  5 = Rooftop or terrace 1 = Yes; 2 = No  6 = Green zones or common-property zones 1 = Yes ; 2 = No |  |  |
| C5 | Show me your bathroom, OBSERVE AND WRITE DOWN WHAT KIND OF TOILET SERVICE DOES THE HOUSE HAVE?  1 = Toilet connected to sewerage  2 = toilet connected to septic tank  3 = Non-connected toilet  4 = Latrine (black hole, pit)  5 = Does not have any toilet | | |
| C6 | Which of the following public, private, or community utility services does your house have?  1= Power 1 = Yes 2 = No  2 = Natural gas connected to a public web? 1 = Yes 2 = No  3 = Aqueduct, water supply? 1 = Yes 2 = No  4 = Sewerage? 1 = Yes 2 = No  5 = Waste collection? 1 = Yes 2 = No | | |
| C7 | \| 1= Radio 1 = Yes 2 = No \| \| --- \| \| 2= Color TV 1 = Yes 2 = No \| \| 3 = Washing machine 1 = Yes 2 = No \| \| 4 = DVD 1 = Yes 2 = No \| \| 5 = Computer 1 = Yes 2 = No \| \| 6 = Working internet 1 = Yes 2 = No \| \| 7 = Air conditioning 1 = Yes 2 = No \| \| 8 = Fan 1 = Yes 2 = No \| | | |
| C8 | Which are the three sources of information you use more to know about what is going on every day in your country? Surveyor: DOES NOT READ THE ANSWERS, ONLY REPORT WHAT PEOPLE ANSWER SPONTANEOUSLY  1 = relatives, friends, and neighbors 2 = community informative  3 = local or community newspaper 4 = national newspaper  5 = radio 6 = television  7 = groups or associations 8 = work or business partners  9 = community leaders 10 = a government official  11 = NGOs 12 = internet | | |

Da. (Have you heard about dengue? Yes No (If no, go to I)

Db. In past 2 years, is anyone infected with dengue at your house? Yes No

Dc. In past 2 years, is anyone infected with dengue in your neighbour? Yes No

| **S.N** | **Variables** | **Yes** | **No** | **Don’t know** |
| --- | --- | --- | --- | --- |
| **Knowledge of symptoms** | | | | |
| D1 | Is fever a symptom of dengue? | 1 | 2 | 3 |
| D2 | Is headache a symptom of dengue fever? | 1 | 2 | 3 |
| D3 | Is joint pain a symptom of dengue fever? | 1 | 2 | 3 |
| D4 | Is muscle pain a symptom of dengue fever? | 1 | 2 | 3 |
| D5 | Is pain behind the eyes a symptom of dengue fever? | 1 | 2 | 3 |
| D6 | Are nausea/vomiting symptoms of dengue fever? | 1 | 2 | 3 |
| D7 | Is rash a symptom of dengue fever? | 1 | 2 | 3 |
| D8 | Is diarrhea common in dengue fever? | 1 | 2 | 3 |
| D9 | Is back pain common in dengue fever? | 1 | 2 | 3 |
| D10 | Is stomach pain common in dengue fever? | 1 | 2 | 3 |
| **Knowledge of transmission** | | | | |
| D11 | Can all mosquitoes transmit dengue fever? | 1 | 2 | 3 |
| D12 | Do the *Aedes* mosquitoes transmit dengue fever? | 1 | 2 | 3 |
| D13 | Do flies transmit Dengue fever? | 1 | 2 | 3 |
| D14 | Do ticks transmit Dengue fever? | 1 | 2 | 3 |
| D15 | Does ordinary person to person contact transmit Dengue fever? | 1 | 2 | 3 |
| D16 | Is Dengue fever transmitted through food and water? | 1 | 2 | 3 |
| D17 | Can dengue fever be transmitted by blood transfusion? | 1 | 2 | 3 |
| D18 | When are the Dengue mosquitoes likely to feed/bite?  1 Night time 2 Day time 3 Both day and night 4 Morning 5 Evening 6 Anytime  7 Dont know |  |  |  |
| D19 | Mosquitoes breed in standing water | 1 | 2 | 3 |
| D20 | Window screens and bed net reduce mosquitoes | 1 | 2 | 3 |
| D21 | Insecticide sprays reduce mosquitoes and prevent Dengue | 1 | 2 | 3 |
| D22 | Tightly covering water containers reduces mosquitoes | 1 | 2 | 3 |
| D23 | Removal of standing water can prevent mosquito breeding | 1 | 2 | 3 |
| D24 | Mosquito repellents prevent mosquitoes | 1 | 2 | 3 |
| **Sources of Information on dengue** | | | | |
| D25a | Radio | 1 | 2 | 3 |
| D25b | Television | 1 | 2 | 3 |
| D25c | Health Professionals | 1 | 2 | 3 |
| D25d | Miking | 1 | 2 | 3 |
| D25e | Neighbors | 1 | 2 | 3 |
| D25f | Teachers | 1 | 2 | 3 |
| D25g | Children | 1 | 2 | 3 |
| D25h | Others ========================== | 1 | 2 | 3 |

**E= Attitudes towards dengue**

| **S.N** | **Attitudes towards dengue** | **Strongly agree** | **Agree** | **Not sure** | **Disagree** | **Strongly Disagree** |
| --- | --- | --- | --- | --- | --- | --- |
| E1 | Dengue fever is not a serious illness? | 1 | 2 | 3 | 4 | 5 |
| E2 | Are you at risk of getting dengue? | 1 | 2 | 3 | 4 | 5 |
| E3 | Dengue fever be prevented? | 1 | 2 | 3 | 4 | 5 |
| E4 | Is controlling the breeding places of mosquitoes a good strategy to prevent dengue fever? | 1 | 2 | 3 | 4 | 5 |
| E5 | Do you think that stagnant water around the houses in discarded tires, broken pots and bottles are breeding places of *Aedes* mosquitoes?) | 1 | 2 | 3 | 4 | 5 |
| E6 | Do you think communities should actively participate in controlling the vectors of Dengue? | 1 | 2 | 3 | 4 | 5 |

**F. PRACTICES IN DENGUE FEVER AND THE VECTOR**

| **S.N** | **Variables** | **Yes** | **No** | **Dont know** |
| --- | --- | --- | --- | --- |
| F1 | Prevent mosquito-man contact | 1 | 2 | 3 |
| F2 | Use insecticide sprays to reduce mosquitoes | 1 | 2 | 3 |
| F3 | Use professional pest control to reduce mosquitoes | 1 | 2 | 3 |
| F4 | Use screen windows to reduce mosquitoes | 1 | 2 | 3 |
| F5 | Eliminate standing water around the house to reduce mosquitoes | 1 | 2 | 3 |
| F6 | Cut down bushes in the yard to reduce mosquitoes | 1 | 2 | 3 |
| F7 | Prevent water stagnation | 1 | 2 | 3 |
| F8 | Use mosquito eating fish to reduce mosquitoes | 1 | 2 | 3 |
| F9 | Use mosquito coils to reduce mosquitoes | 1 | 2 | 3 |
| F10 | Cleaning of garbage/ trash | 1 | 2 | 3 |
| F11 | Disposing water holding containers such as tires, parts of automobiles, plastic bottles, crack pots etc.) | 1 | 2 | 3 |
| F12 | Use Mosquito Repellent/ cream | 1 | 2 | 3 |
| H13 | Use of fan | 1 | 2 | 3 |
| F14 | Use of smoke to drive away mosquitoes | 1 | 2 | 3 |
| F15 | Covering body with clothes | 1 | 2 | 3 |
| F16 | Do nothing to reduce mosquitoes | 1 | 2 | 3 |
| F17 | Eliminating mosquito breeding sites | 1 | 2 | 3 |
| F18 | Cover water containers in the home | 1 | 2 | 3 |
| F19 | Frequently cleaning water filled containers and ditches around the house )  *1 Always* | 1 | 2 | 3 |
|  | *2 Often*  *3 Sometimes*  *4 Never*  *5 Dont know* |  |  |  |
| F20 | Government sprays insecticides for controlling mosquitoes | 1 | 2 | 3 |
| F21 | Turning containers upside down to avoid water collection | 1 | 2 | 3 |
